# Anomalous Nernst Effect-Based Near-Field Imaging of Magnetic Nanostructures

**DOI:** 10.1021/acsnano.4c09749

**Published:** 2024-11-05

**Authors:** Atul Pandey, Jitul Deka, Jiho Yoon, Anagha Mathew, Chris Koerner, Rouven Dreyer, James M. Taylor, Stuart S. P. Parkin, Georg Woltersdorf

**Affiliations:** † Max Planck Institute of Microstructure Physics, Weinberg 2, Halle 06120, Germany; ‡ Institute of Physics, Martin Luther University Halle-Wittenberg, Von-Danckelmann-Platz 3, Halle 06120, Germany

**Keywords:** anomalous Nernst effect, magnetic textures, magnetic domains, nanostructures, scanning probe microscopy, optical near field, thermal gradient

## Abstract

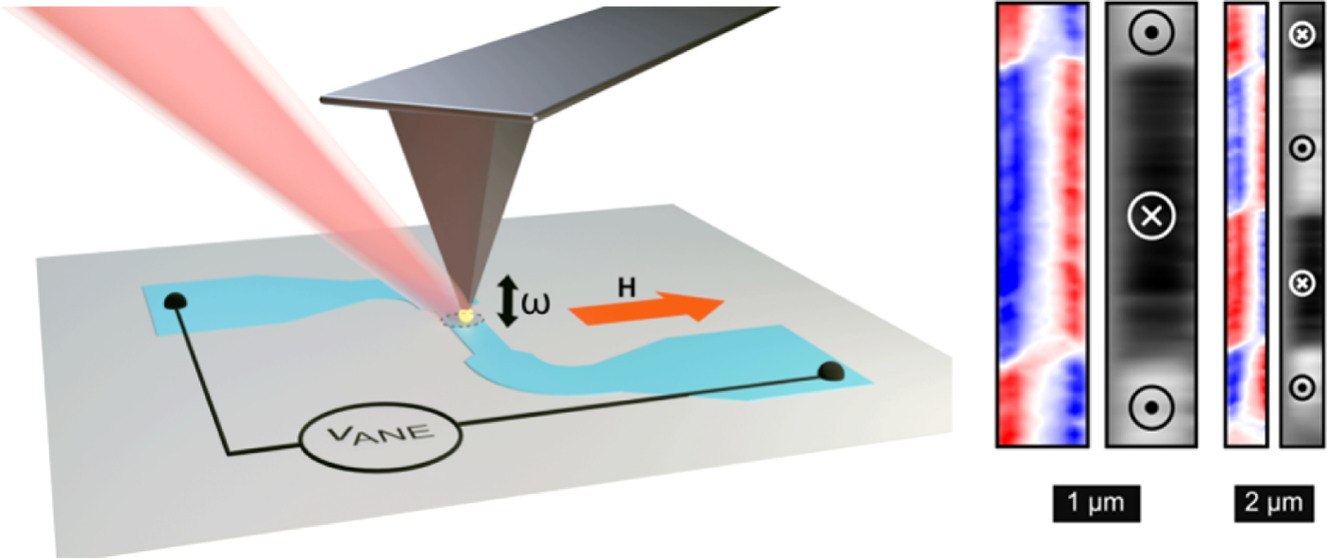

The anomalous Nernst effect (ANE) gives rise to an electrical response transverse to magnetization and an applied temperature gradient in a magnetic metal. A nanoscale temperature gradient can be generated by the use of a laser beam applied to the apex of an atomic force microscope tip, thereby allowing for spatially resolved ANE measurements beyond the optical diffraction limit. Such a method has been previously used to map in-plane magnetized magnetic textures. However, the spatial distribution of the out-of-plane temperature gradient, which is needed to fully interpret such ANE-based imaging, was not studied. We therefore use a well-known magnetic texture, a magnetic vortex core, to demonstrate the reliability of the ANE method for imaging of magnetic domains with nanoscale resolution. Moreover, since the ANE signal is directly proportional to the temperature gradient, we can also consider the inverse problem and deduce information about the nanoscale temperature distribution. Our results together with finite element modeling indicate that besides the out-of-plane temperature gradients there are even larger in-plane temperature gradients. Thus, we extend the ANE imaging to study the out-of-plane magnetization in a racetrack nanowire by detecting the ANE signal generated by in-plane temperature gradients. In all cases, a spatial resolution of ≈70 nm is obtained. These results are significant for the rapidly growing field of thermoelectric imaging of antiferromagnetic spintronic device structures.

## 1 Introduction

The miniaturization of spintronic devices requires individual magnetic entities to be densely packed. Magnetic stray fields due to the interactions between neighboring bits are a major limitation for the packing density when nanoscale ferromagnets are used.^1,2^ An elegant solution that has been used since the very first spintronic sensors, as well as in magnetic random access memories, is a synthetic antiferromagnet^3^ formed from thin ferromagnetic layers coupled via a thin metallic antiferromagnetic coupling layer.

Recently, there has been increased interest in utilizing innately antiferromagnetic (AF) materials for spintronic applications,^2,4−6^ which are free from stray fields. The absence of stray fields makes it very difficult to both calculate the size and to image AF domains. Typically, it is understood that these materials exhibit domain structures at the submicrometer scale.^7,8^ In order to understand their behavior (e.g., their response to magnetic fields and spin torques or interaction with structural defects) imaging of the magnetic order with nanometer resolution is required. Magneto-optical imaging techniques are limited in their resolution to the wavelength of the light,^9^ while X-ray based photoemission microscopy is extremely surface sensitive.^10−13^

The magnetic state of ferromagnetic materials can be inferred from their off-diagonal magnetotransport behavior.^14,15^ In analogy with ferromagnets, whose magnetization is a symmetry-breaking order parameter, the magnetic state of antiferromagnets with a magnetic octupole order parameter can be inferred from the off-diagonal elements of their Berry curvature-driven magnetotransport.^16−19^ One important example is the anomalous Nernst effect (ANE) that generates an electric field (**E**) transverse to both the magnetization (**M**) and an applied temperature gradient (*T*).^20^

By using laser heating to generate a local *T* that can be rastered across the sample, spatially resolved measurements of ANE-generated voltage (*V*_ANE_) allow for the imaging of magnetic domains in both ferromagnets and antiferromagnets. This method of scanning ANE (SANE) microscopy has previously been used to image the magnetic domains exhibited by in-plane (IP) magnetized thin-film devices with a spatial resolution of a few micrometers.^21−23^ More recently, the spatial resolution has been improved by using a nanoscale metallic tip as a near-field antenna.^24−26^ In practice, a metallized tip of an atomic force microscope (AFM) is used to confine the laser heating to a nanoscale region under the tip.

Here, we build on the previous work to extend ANE-based near-field microscopy (NF-SANE) to out-of-plane magnetization imaging. Based on finite element simulations of the laser heating on the nanoscale, we find that the in-plane component of the temperature gradient,^24,27,28^ is actually twice as large as its out-of-plane (OOP) component. Inspired by this result, we first image the well-known IP-magnetized Landau pattern of a magnetic vortex state using the ANE signal generated by the OOP temperature gradient. This allows us to demonstrate the validity of the ANE microscope principle and compute the spatial distribution of the OOP temperature gradient. In the second step, the magnetic domains in an OOP magnetized racetrack nanowire are imaged by using the ANE signal resulting from an IP temperature gradient. In both cases, the spatial resolution we obtain is ≈70 nm.

## 2 Results

The electrical response resulting from the ANE is given by , where *S*_ANE_ is the ANE coefficient. The resulting voltage measured along the device length (*y*-direction) is described by the following equation:
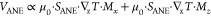
1

**Figure 1:**
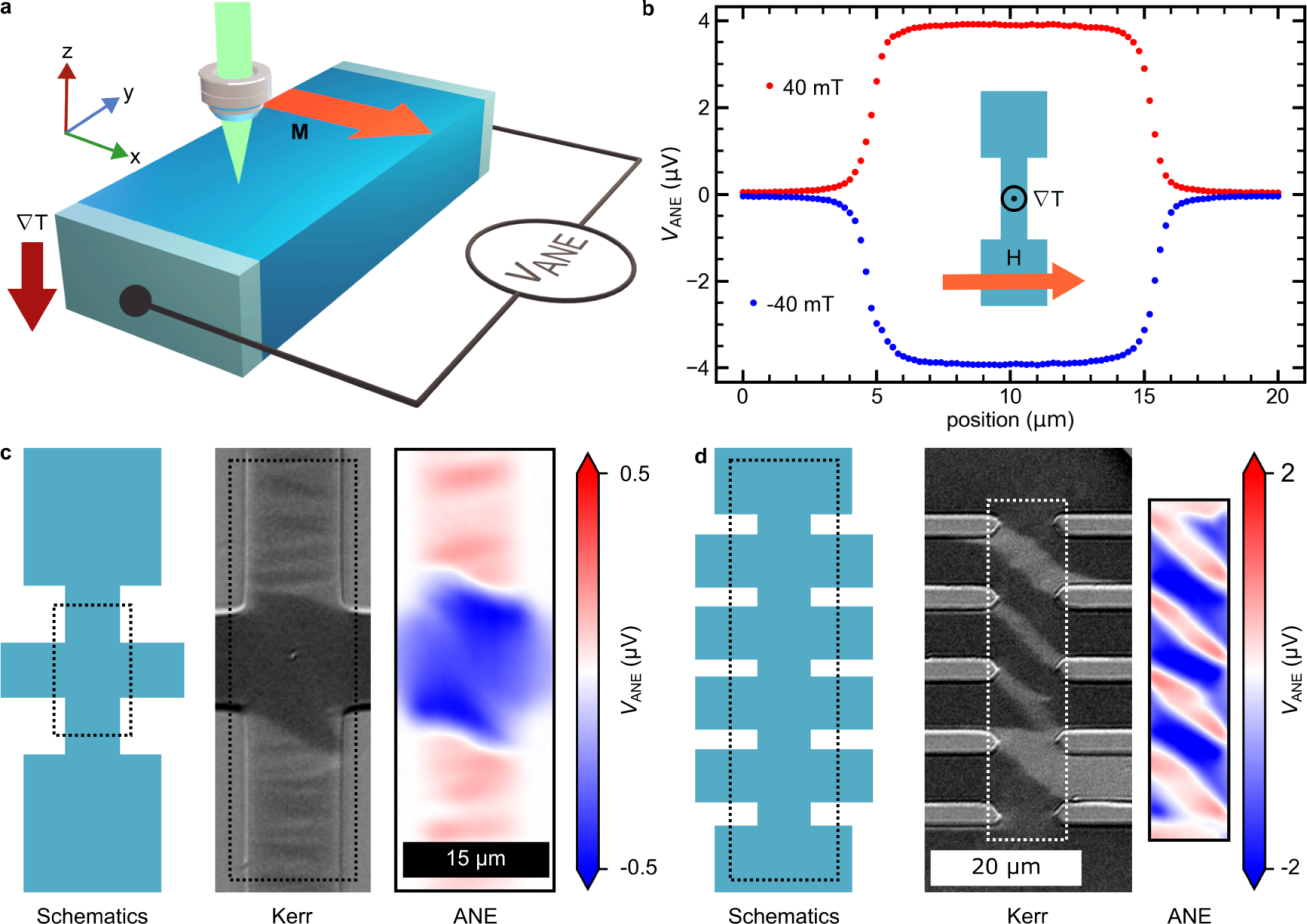
(a) Schematic illustration of the ANE imaging method. The *V*_ANE_ is given by the transverse component of the magnetization shown by the orange arrow and the vertical temperature gradient shown by the red arrow. (b) Line scan of *V*_ANE_ across a device of width *w* = 10 μm in a field of 40 and −40 mT. The device is illuminated by a 5 mW laser beam with a wavelength of 532 nm focused by a 60× (NA = 0.7) objective. The inset shows a schematic of the device structure utilized for the measurements. (c,d) Kerr and SANE microscopy images of multidomain states stabilized in different device structures as indicated with the schematics.

In order to validate the applicability of the ANE microscopy to image magnetic textures we verify the magnetic origin of the laser-induced signals and compare magneto-optic microscopy and SANE microscopy^21−23^ images of magnetic microstructures with domains. Here, we use a laser beam focused by a microscope objective to create a temperature gradient while scanning the sample laterally for imaging (Figure 1a). A 15 nm thick IP-magnetized Co_20_Fe_60_B_20_ film is patterned into a 10 μm wide wire as shown with the schematic (Figure 1b). A magnetic field μ_0_H is applied along the width of the wire. We observe a *V*_ANE_ signal on the order of a few μV as the laser beam is scanned across the wire. This signal clearly is of magnetic origin as its polarity changes when the magnetic field is reversed in the direction.

Next, we show that spatially resolved ANE measurements can be utilized to image the magnetic domain structure of a sample. For this, we use similar 15 nm thick CoFeB wire structures with branches, as shown in Figure 1c,d. These branches stabilize a multidomain remnant state, as confirmed by static Kerr microscopy. The corresponding ANE measurements reproduce these domain structures well with much better contrast.

**Figure 2:**
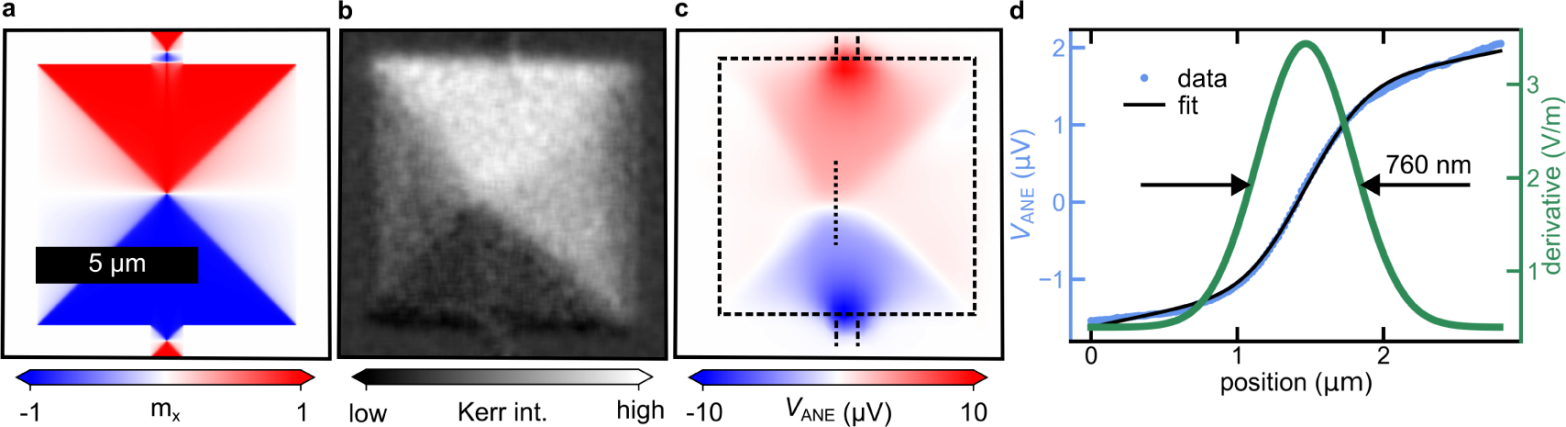
(a) Micromagnetic simulation of an 8 × 8 μm^2^ CoFeB slab of 45 nm thickness fashioned with 1 μm wide contact electrodes at the top and bottom. (b) Longitudinal Kerr intensity image showing the *x* component of the magnetization of the film. (c) ANE microscope image of the magnetic domains in the same device as (b), the outline of which is shown by the dashed box. (d) Blue data points (left axis) show the ANE signal measured in a line scan across the center of the vortex, as indicated by the dotted line shown in (c). This line scan data are fitted with an error function with a linear background (black line). The derivative of the fitted error function is then plotted as a green line (right axis).

Applying this method to a nanoscale spin texture of a magnetic vortex^29−31^ allows us to understand the spatial spreading of the thermal heat gradients. For this, we use a 8 × 8 μm^2^ CoFeB slab of 45 nm thickness, with 1 μm wide contact wires (top and bottom in the image in Figure 2. The ANE signal, along the *y*-direction that is proportional to *m*_*x*_, is measured using these contacts.

A differential Kerr microscope image, as shown in Figure 2b, reveals the expected pattern around the vortex core of this structure. SANE imaging reproduces this pattern well, as demonstrated in Figure 2c. We note that the ANE signal is higher near the wire connections. This is due to the lateral spreading of the current generated by V_ANE_ throughout the slab.

A magnetic vortex structure leads to a very rapid rotation of the IP magnetization as the vortex core has a width of only a few nm.^29−31^ This results in a nanoscale transition between opposing in-plane magnetization directions across the vortex. An ANE line scan through the vortex allows us to compute the spatial distribution of the heat gradient Section S8), which is given by the derivative of the ANE line scan. The ANE line scan is well described by an error function summed with a linear term (Figure 2d). The error function accounts for change in the ANE signal at the domain wall, while the linear term reflects the variation of the signal strength due to current distribution. The spatial distribution of  is obtained by computing the derivative of the fit to ANE line scan, which is a Gaussian with a full width at half-maximum (FWHM) of 760 ± 18 nm. This gradient distribution is very close to the intensity distribution of the focused laser beam used for SANE, which is also a Gaussian with a FWHM = 736 ± 22 nm (Figure S7b). Finite-element modeling analysis^32^ reveals that the OOP temperature gradient closely follows the radial distribution of the laser intensity (Figure S5d). This is consistent with our findings.

Next, we test if this is still the case on the nanoscale, i.e., when optical near-field focusing near the apex of a metallic AFM tip^33,34^ confines the heat source to ≈20 nm. Our simulations show that even for a 20 nm wide Gaussian heat source the broadening of the heat gradient due to thermal spreading in the sample only amounts to ≈32%, as shown in (Figure S5d). This implies that heat spreading has only a small effect on the spatial resolution, even for NF-SANE imaging.

**Figure 3:**
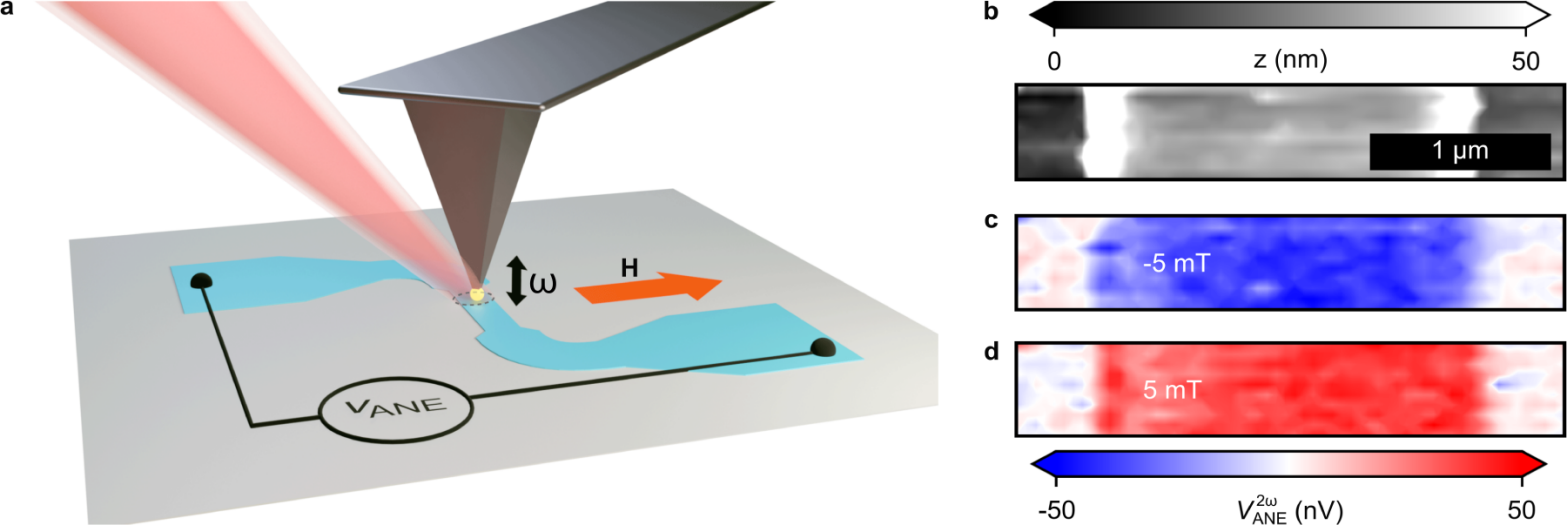
(a) Schematic illustration of the NF-SANE microscope. A laser beam with a wavelength of 8 μm and a power of 25 mW is focused at the apex of an AFM tip. The ANE signal generated by the enhanced near-field underneath the tip apex is detected. (b) AFM topographic scan of a 2 μm wide magnetic wire. (c,d) ANE 2^nd^ harmonic signal under the application of a transverse 5 mT and −5 mT magnetic field, respectively.

Thus, we now experimentally study ANE imaging with nanoscale gradients created by the enhanced optical near-field^35,36^ of a metallic AFM tip (Figure 3a). The tapping of the AFM tip generates an intensity-modulated optical near field that, in turn, generates an oscillating thermal gradient with a radial distribution on the nanometer scale. The enhanced optical near field at the sample has a nonlinear dependence on the tip-to-sample distance. Therefore, the sinusoidal oscillation of the AFM tip results in the generation of higher harmonics of the tip oscillation frequency in the ANE signal. We record the voltage demodulated at the second harmonic of the tip oscillation frequency in order to detect the ANE signal that exclusively originates from the optical near field. In the experiment, we again used a 15 nm thick CoFeB film now patterned into a narrower 2 μm wide wire, as shown by the AFM image in Figure 3b. A magnetic field of ±5 mT is applied along the *x*-direction of the wire structure. The resulting NF-SANE images for both field directions are shown in Figure 3c,d respectively. When the tip approaches the magnetic wire, an ANE signal on the order of 40 nV is observed. Again, the voltage polarity reverses with the field direction, confirming the magnetic origin of the near-field signal.

**Figure 4:**
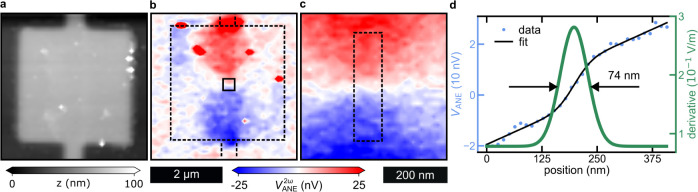
(a) AFM height scan of a 3 × 3 μm^2^ square device. (b) Simultaneously measured 2nd-harmonic ANE voltage. Outer dashed square indicates the edge of the device. (c) High-resolution ANE scan in an area around the magnetic vortex shown by the inner solid square in (b). (d) Average ANE signal from eight line scans measured in the dotted rectangle region shown in (c) (Blue data points (left axis) show the ANE signal measured in a line scan across the center of the vortex, as indicated by the dotted line in (c). This line scan data is fitted with an error function with a linear background (black line). The derivative of the fitted error function is then plotted as a green line (right axis).

To study the nanoscale heat gradient, we again use a Landau pattern but on a smaller device consisting of a 3 × 3 μm^2^ square connected to 500 nm wide contact wires. An AFM height scan with a 100 nm step size shows the overall structure of the device in Figure 4a. The simultaneously measured ANE signal in Figure 4b reveals a Landau pattern, representing the magnetic domain structure. A scan with a smaller step size (12 nm) shows the vortex core region with higher resolution, Figure 4c.

In Figure 4d we follow the same procedure as in Figure 2d and show the corresponding heat gradient distribution for the NF-SANE case. Again, we find a Gaussian distribution with an FWHM of 74 ± 14 nm. In such a scanning measurement, the resolution is determined by the FWHM of the probe. i.e., we can expect a resolution of ≈74 nm for NF-SANE.

These measurements of a Landau pattern rely on OOP temperature gradient , which was previously assumed to be the only relevant contribution.^26^ However, our finite element simulations of the heat distribution show that the IP temperature gradient () is almost a factor of 2 larger than the OOP temperature gradient (Figure S6). This is in line with previous observations, where this gradient had been used to probe OOP magnetization.^24,27,28^ We aim to quantify the utility of the IP gradient by using a well-defined OOP magnetic structure. In doing so, we want to resolve the contradiction regarding the relative magnitudes of IP and OOP temperature gradients and show how it can be utilized to visualize OOP magnetization on the nanoscale.

The near-field focus of light leads to a spatially nonuniform IP temperature gradient  (Figure S6). In this case, the second term in eq 1 can be written as follows (Section S7):
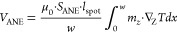
2where *l* and *w* correspond to the length and width of the wire, respectively, and *l*_spot_ is the spot size of the heat gradient. For a uniform OOP magnetization under a heat source, the eq 2 can be simplified as follows:
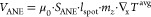
3Where  is the average IP temperature gradient in the wire.

**Figure 5:**
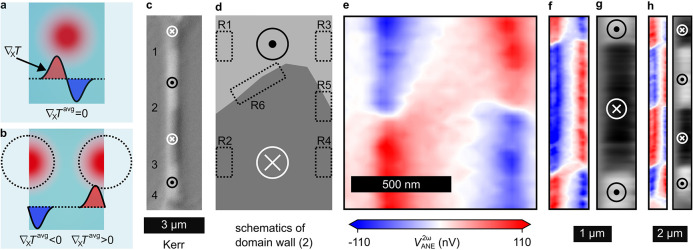
(a) Schematic showing an OOP magnetic film on a transparent substrate. The laser illumination creates an asymmetric IP temperature gradient resulting in zero . (b) Schematic showing a laser beam focused near the edges of a metallic film that partially illuminates the film. In this case,  is nonzero and of opposite sign at the two edges. (c) Polar Kerr microscope image of the racetrack nanowire. The numbers indicate the different domain walls. (d) Schematics showing different regions around the magnetic domain wall #2. (e) Near-field-based ANE microscope image of the area surrounding the domain wall #2. (f) Same as (e) for larger area consisting of domain walls #2 and #3. (g) ANE signal in (f) is integrated along the *x*-direction. (h) Same as (f,g) for the ANE measurement performed across the full nanowire consisting of four domain walls.

At the edges of the sample, a nonzero  arises, as illustrated in Figure 5a,b. Hence, one can expect that at the edges of an OOP magnetized sample, a large ANE signal will be generated, while it should vanish in the center of the wire due to a vanishing . In addition, even for the case of zero  in the center region of the wire, a nonuniform OOP magnetization (e.g., close to a domain wall) will result in a finite ANE signal because the two lobes of the IP thermal gradient no longer cancel. This can be seen from eq 2. We now demonstrate the validity of these conclusions by NF-SANE microscopy using a Co/Ni multilayer stack with perpendicular magnetic anisotropy.^37,38^ The Co/Ni stack is patterned into a 700 nm wide nanowire. A multidomain state is induced by using a decaying AC OOP magnetic field. The prepared sample consists of four domain walls within the narrow section of the wire, as confirmed by polar Kerr microscopy (Figure 5c). Subsequently, this magnetic structure is investigated with NF-SANE.

In agreement with our prediction of , NF-SANE indeed shows large signals for the OOP-magnetized sample that are related to the domain state. We now analyze this result in detail. We focus on the second domain wall in Figure 5c, as schematically shown in Figure 5d. Here, different regions of interest are marked as R1–R6. R1 and R2 at the left edge are characterized by uniform *m*_*z*_^up^ and *m*_*z*_^down^. The negative  at this edge generates a negative V_ANE_ for *m*_*z*_^up^ in R1, and a positive V_ANE_ for *m*_*z*_^down^ in R2, as shown in Figure 5e. Similarly, a positive  at the right edge results in a positive V_ANE_ for *m*_*z*_^up^ in region R3 and a negative V_ANE_ for *m*_*z*_^down^ in R4. These findings demonstrate that the ANE near-field signal is sensitive to the OOP magnetization in the vicinity of the edges.

Next, we consider region R5, which consists of a magnetic domain wall at the right edge of the wire. We performed a line scan in the *y*-direction. Since the ANE signal in this region is proportional to the magnetization, we can obtain the spatial resolution of the ANE microscope with such a line scan across the domain wall. We thereby find a spatial resolution of 56 ± 10 nm (Figure S10b). Thus, we are able to employ NF-SANE to image domain walls in OOP-magnetized structures with a resolution that is better than 60 nm.

R6 consists of a domain wall in the middle of the wire. Illuminating this region creates a positive  on the left half of the laser spot. This generates a positive V_ANE_ with a value of *m*_*z*_^up^. The right half of the laser spot creates a negative  that also generates a positive V_ANE_ with *m*_*z*_^down^. Thus,  and  on either side of the domain wall generate the same sign of voltage resulting in a net positive ANE signal, as we observe at the upper domain wall in Figure 5f. Similarly, a negative ANE voltage is observed at the lower domain wall because in this case the positive and negative  illuminate *m*_*z*_^down^ and *m*_*z*_^up^ domains, respectively. The vicinity of domain walls in PMA materials is characterized by a large lateral gradient of the OOP magnetization. This explains the observed domain wall contrast in the ANE signals.

Domain walls of the OOP-magnetized materials also contain regions with IP magnetization. However, the domain wall width is expected to be only ≈5 nm in our Co/Ni sample.^37^ Thus, underneath the ≈60 nm wide heat gradient spot, the ANE signal from the IP-magnetized states would be expected to be smaller by at least one order of the magnitude. Based on this, the ANE signal should be dominated by the OOP magnetization in our measurements.

Determining the magnetic structure in nanowires is perhaps the most important application of the NF-SANE imaging method. This specific geometry allows one to numerically integrate the observed ANE signal across the wire width to obtain a signal that is proportional to the magnetization and not only its gradient. The result of this procedure is shown in the gray scale images Figure 5g,h directly reflecting the domain structure in the wire.

## 3 Discussion

We discuss the underlying mechanisms that make ANE microscopy advantageous when studying magnetic materials on the nanoscale. The magnitude of the ANE voltage in a given magnetic wire is proportional to  (Section S4), where *P* is the total absorbed power that contributes to heating the wire, and *w* is the wire width. The magnitude of the ANE signal is independent of all of the other geometric factors. The laser-based heating utilized in ANE microscopy has two advantages in this regard. First, the entire absorbed energy is utilized to directly heat the wire, in contrast to resistive-heating methods where a majority of the heat energy is dissipated elsewhere.^39−41^ Second, since the voltage is inversely proportional to the wire width, studying narrower wires with ANE microscopy gives rise to larger signals.

In addition, this result () allows us to understand the magnitude of the nanoscale plasmonic enhancement of the optical field below the AFM tip.^35,36^ For this, we compared the ANE signals obtained by means of SANE and NF-SANE using wires composed of the same material. We observe *V*_ANE_ = 4 μV in a 10 μm wide wire induced by a (5 mW × 0.35 = 1.75 mW) focused laser beam, taking the reflection coefficient of the CoFeB film to be 0.65 for the laser beam with a wavelength of 532 nm.^42^ Based on this, our observation of a 40 nV signal with the NF-SANE signal in a 2 μm wide wire (see, for example, Figure 3) would require a laser power of 3.5 × 10^–3^ mW underneath the AFM tip. In our NF-SANE setup, a 25 mW laser beam is focused by a 0.4 nm numerical aperture parabolic mirror. This illuminates the tip over a circular region of diameter 10 μm, resulting in 1.6 × 10^–3^ mW at the 80 nm wide laser spot underneath the tip. Considering a reflection coefficient of 0.95 for the given laser beam with a wavelength of 8 μm,^42^ the absorbed laser power amounts to only 8 × 10^–5^ mW. This means that the near-field interaction between the AFM tip and the sample enhances the laser power underneath the tip by a factor of approximately 44.

We obtain OOP temperature gradient to be a Gaussian distribution with an amplitude of ≈6 × 10^6^ K/m and ≈12 × 10^6^ K/m for SANE and NF-SANE cases, respectively (Section S9).

Finally, we discuss our findings in the context of rapidly developing research on antiferromagnetic materials. Since ANE is a Berry curvature-driven response that is present in conducting chiral antiferromagnets,^22,26^ our results concerning the IP and OOP sensitivity of the ANE method are directly applicable to study spatially resolved switching in these systems and sense the spin orientation.^43^ This contrasts the pure OOP sensitivity of anomalous Hall effect experiments usually employed for electrical detection of the magnetic configuration in chiral antiferromagnets^16,17^

## 4 Conclusions

We have demonstrated the reliability of ANE microscopy to image magnetic domain states in in-plane magnetized wires down to the nanoscale. Our analysis also reveals the presence of a very large in-plane gradient that can be employed to probe out-of-plane magnetization and magnetic domain walls with a resolution of 70 nm, most suitable for racetrack nano wires. In addition, by using well-known magnetic textures, such as a vortex or a domain wall, one can analyze the spatial distribution of the temperature gradients at the nanoscale.

## 5 Methods

### 5.1 The Film Deposition and the Device Fabrication

The CoFeB films with thicknesses of 15 and 45 nm were deposited using an ultrahigh vacuum magnetron sputtering system on (001)-cut MgO substrates. The films were capped with a 2 nm alumina protective layer. The base pressure of the sputtering system is 3 × 10^–8^ mbar, while the Ar process gas pressure during deposition was 4.5 × 10^–3^ mbar. The deposition rate was 0.1 Å/s.

The sample with out-of-plane magnetization had the following layer structure: TaN(50)/Pt(12)/Co(3)/Ni(7)/Co(2)/Ni(7)/Co(2)/Ni(7)/Co(2)/TaN(30) (numbers represent the thicknesses in angstroms). These were deposited on sapphire substrates using a second ultrahigh vacuum magnetron sputtering system with a base pressure of 3 × 10^–9^ mbar, while the Ar process gas pressure was 3 × 10^–3^ mbar. Pt, Co, and Ni were deposited at rates of 0.82, 0.21, and 0.22 Å/s, respectively. All the deposition steps were performed at room temperature.

The wire structures for the CoFeB samples were defined by using lithography and lift-off steps. Electron beam lithography was used for the devices with features smaller than 2 μm, while the devices with larger structures were patterned using a maskless optical lithography system. The out-of-plane magnetized Co/Ni stack was patterned by using e-beam lithography and dry etching by using Ar-ion bombardment.

### 5.2 Scanning ANE Microscope Setup

For spatially resolved scanning ANE measurements, a laser beam with a wavelength of 532 nm is focused by an objective lens with a numerical aperture of 0.7 to create a localized temperature gradient. The sample underneath the focused laser spot is scanned with a piezo stage. In order to modulate the laser heating induced ANE signal, the laser beam is modulated with an optical chopper at a frequency of 600 Hz. The resulting ANE signal is detected by demodulating the measured voltage with a lock-in amplifier.

### 5.3 Near-Field Scanning ANE Microscope

For the NF-SANE setup, a commercial scanning near-field optical microscope (SNOM) from the company Attocube/Neaspec was utilized. A laser beam with a wavelength of 8 μm was focused on a Pt-Ir tip that has a resonance frequency of ≈280 kHz. The generated ANE signal was demodulated at the second harmonic of the tip vibration frequency by using a lock-in amplifier. The samples were mounted into the SNOM using a home-built sample holder with a small magnet. No additional preamplifier were used for detecting the measured ANE signal.

## Data Availability

All primary data that support our findings of this study, as well as the codes used for the analysis are available at Zenodo (https://doi.org/10.5281/zenodo.13947405).

## Supporting Information Available

Schematics of the experimental setup for the scanning ANE microscope; dependence of the ANE signal magnitude on input laser power; dependence of the ANE signal magnitude on width of the device wire structures; dependence of the ANE signal magnitude on the area of the input heat flux; COMSOL simulations for out-of-plane heat gradient; COMSOL simulations for in-plane heat gradient; intensity distribution of the focused laser beam; spatial distribution of the out-of-plane temperature gradient; spatial distribution of the in-plane temperature gradient; micromagnetic simulations; ANE-based hysteresis measurement; and magnetic characterization of the OOP-magnetized sample (PDF)
