# Supplementary material for: Anomalous Nernst Effect-Based Near-Field Imaging of Magnetic Nanostructures
Source: ACS Nano. 2024 Nov 5;18(46):31949–56. doi: 10.1021/acsnano.4c09749 (PMC11580384; doi:10.1021/acsnano.4c09749)
Supplement: Supplementary file 1 — nn4c09749_si_001.pdf [file nn4c09749_si_001.pdf]

Supporting Information

for

Anomalous Nernst effect based near field imaging of magnetic nanostructures

Atul Pandey,[†,‡] Jitul Deka,[†] Jiho Yoon,[†] Chris Koerner,[‡] Rouven Dreyer,[‡] James M. Taylor,[‡] Stuart S. P. Parkin,[†] and Georg Woltersdorf[*,‡,†]

†*Max Planck Institute of Microstructure Physics, Weinberg 2, 06120 Halle, Germany*

‡*Institute of Physics, Martin Luther University Halle-Wittenberg, Von-Danckelmann-Platz 3, 06120 Halle, Germany*

E-mail: georg.woltersdorf@physik.uni-halle.de

Contents

S1 ANE microscopy setup **3**

S2 Laser power dependence of the ANE signal **3**

S3 Wire width dependence of the ANE signal **4**

S4 Dependence of ANE signal on heat flux area **5**

S5 Laser heating induced temperature gradient **7**

S6 Intensity distribution of the focused laser beam **10**

S7 Generalised expression for ANE Signal in ANE microscope **11**

S8 Spatial distribution of the temperature gradient **13**

S9 Magnitude of the OOP temperature gradient **14**

S10 NF-SANE spatial resolution with the domain wall in PMA wire **15**

S11 Micromagnetic simulations **15**

S12 Magnetic characterization with SANE microscopy **16**

S13 Magnetic property of the Co/Ni film **17**

S1 ANE microscopy setup

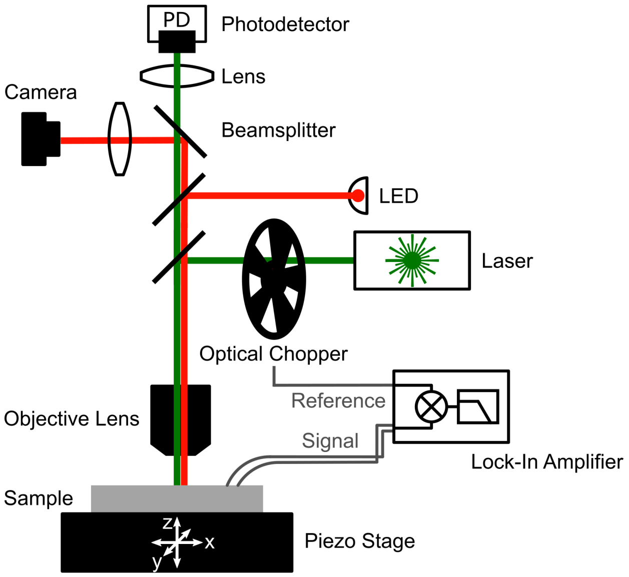

Figure S1: The experimental setup for the scanning ANE (SANE) microscope consists of a 532 nm cw laser beam from a diode-pumped solid-state laser. The beam is focused on the sample by a high numerical aperture objective lens ($NA = 0.7$). The intensity of the laser beam is modulated using an optical chopper. A piezo stage allows to scan the sample under the focal spot while the ANE signal is detected electrically. The intensity of the reflected laser beam can be measured with a photodetector.

S2 Laser power dependence of the ANE signal

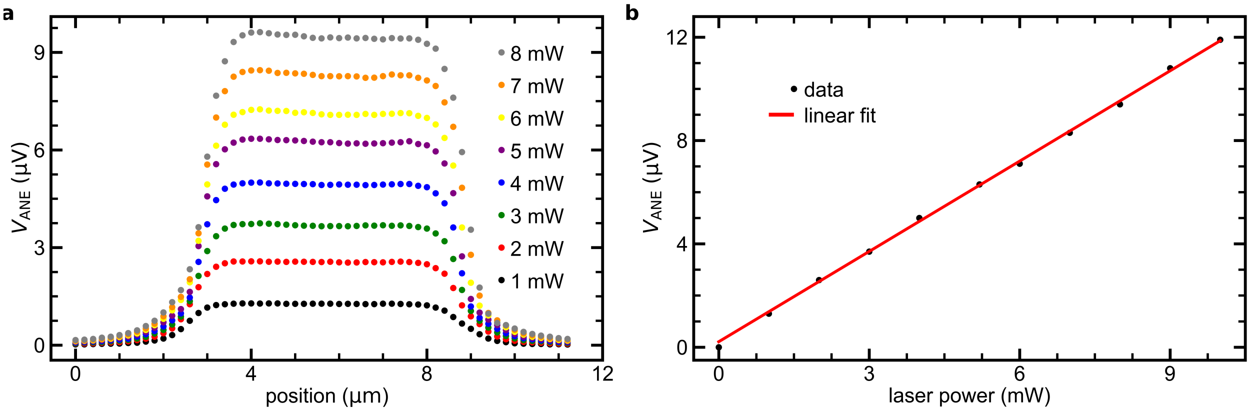

Figure S2: (a) ANE line scan across a 6 µm wide wire at different laser powers. (b) Average ANE signal across the wire width scales linearly with laser power.

S3 Wire width dependence of the ANE signal

Having observed that the magnitude of the ANE signal depends on the wire width when other experimental parameters are kept identical, we developed an analytical model for this dependence and verify the model with the experimental data. We consider the scenario in which a part of the magnetic wire is subjected to a heat flux that creates an out-of-plane (OOP) temperature gradient. This is shown in red in Figure S3a. The illuminated area (of dimension l_{spot}) acts as a battery due to the ANE-induced voltage (V_{gen}). This battery has an internal resistance (R_{int}) due to the finite resistance of the illuminated part of the wire. The regions of the wire shown in green provide a conducting path with resistance R_{c}. The equivalent circuit diagram is shown in Figure S3b, where R_{wire} indicates resistance of the remainder of the magnetic wire as well as the resistance of the connecting leads.

We determine the influence of R_{c} and R_{int} on the magnitude of the measured ANE voltage (V_{meas}). Assuming R_{wire} to be small ($\approx 1\,\mathrm{k}\Omega$) relative to the lock-in impedance, its role can be ignored. Therefore V_{meas} is simply the potential across R_{c}, which is equal to $I \cdot R_{\mathrm{c}}$, where I is the current flowing in the circuit.

$$\begin{aligned} V_{\mathrm{meas}} &= I \cdot R_{\mathrm{c}} \\ &= \frac{V_{\mathrm{gen}}}{R_{\mathrm{c}} + R_{\mathrm{int}}} \cdot R_{\mathrm{c}} \\ &= \frac{R_{\mathrm{c}}}{R_{\mathrm{c}} + R_{\mathrm{int}}} \cdot V_{\mathrm{gen}} \end{aligned}$$

Since $R_{\mathrm{c}} \propto \frac{1}{w - l_{\mathrm{spot}}}$ and $R_{\mathrm{int}} \propto \frac{1}{l_{\mathrm{spot}}}$, where w is the wire width,

$$V_{\mathrm{meas}} = \frac{\frac{1}{w - l_{\mathrm{spot}}}}{\frac{1}{w - l_{\mathrm{spot}}} + \frac{1}{l_{\mathrm{spot}}}} \cdot V_{\mathrm{gen}}$$

$$V_{\mathrm{meas}} = \frac{l_{\mathrm{spot}}}{w} \cdot V_{\mathrm{gen}} \tag{S1}$$

Thus, we find that the measured ANE signal is inversely proportional to the wire width.

ANE line scans across magnetic wires of different widths are shown in Figure S3c. The inverse dependence of V_{ANE} on w is demonstrated in Figure S3d and verified by a linear fit of $1/V_{\mathrm{ANE}}$ against w.

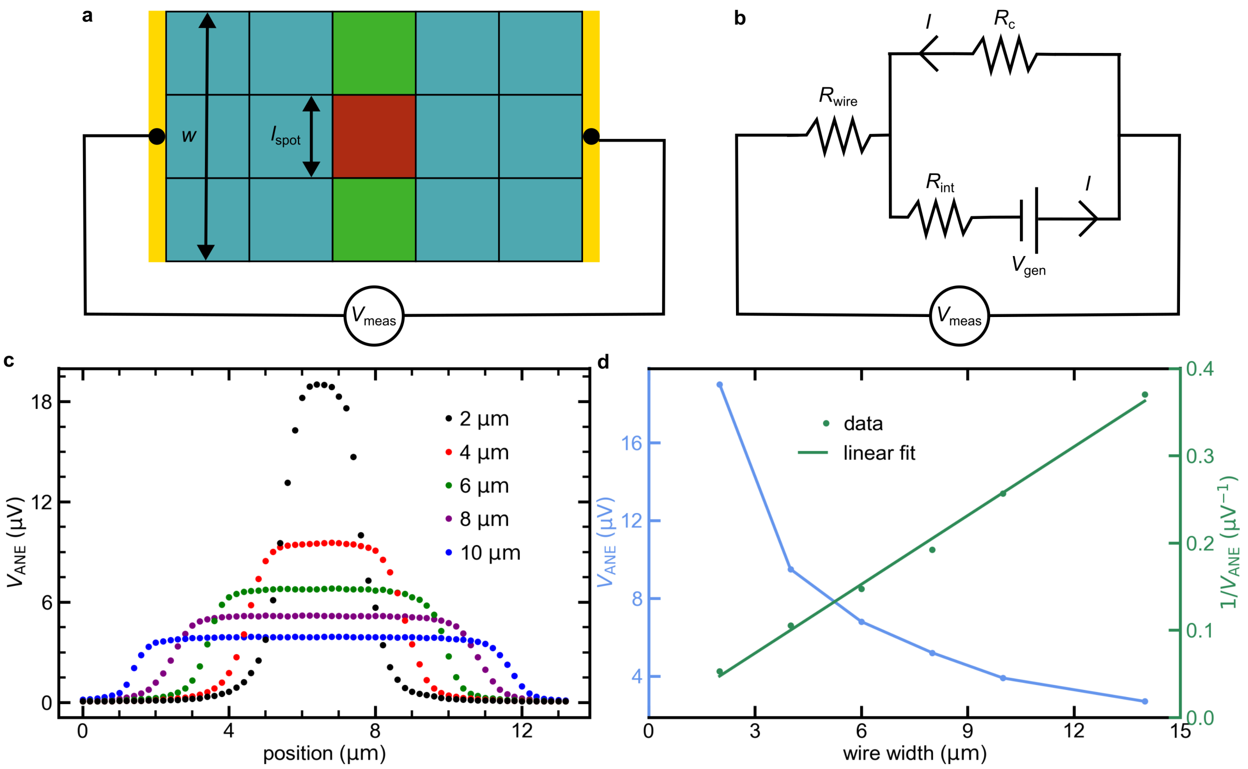

Figure S3: (a) Schematic showing a magnetic wire of width w, with part of the wire across the dimension l_{spot} subjected to a heat flux (b) Equivalent electrical circuit diagram for the device in (a). (c) ANE line scans across magnetic wires of different widths. (d) Average V_{ANE} signal across the magnetic wires plotted against w (blue, left axis) and $1/V_{\mathrm{ANE}}$ plotted against w (green, right axis).

S4 Dependence of ANE signal on heat flux area

We now consider a magnetic wire of width w and thickness t subjected to a heat flux of P in Watts across a $L_{\mathrm{x}} \cdot L_{\mathrm{y}}$ rectangular area (Figure S4a). We determine if L_{x} or L_{y} influences the magnitude of the measured ANE signal V_{ANE}. The heat flux creates an out-of-plane temperature gradient ∇T_{z}. This results in an ANE signal generated along the y-direction ($V_{\mathrm{gen}}^{\mathrm{y}}$). In terms of the ANE coefficient S_{ANE}, it can be written as $V_{\mathrm{gen}}^{\mathrm{y}} = \mu \cdot S_{\mathrm{ANE}} \cdot \nabla T_{\mathrm{z}} \cdot L_{\mathrm{y}}$.

The temperature gradient is proportional to the heat flux density, giving,

$$\begin{aligned} V_{\text{gen}}^{\text{y}} &= \mu \cdot S_{\text{ANE}} \cdot \nabla T_{\text{z}} \cdot L_{\text{y}} \\ &\propto \mu \cdot S_{\text{ANE}} \cdot \frac{P}{L_{\text{x}} L_{\text{y}}} \cdot L_{\text{y}} \\ &\propto \frac{\mu \cdot S_{\text{ANE}} \cdot P}{L_{\text{x}}} \end{aligned}$$

V_{ANE} is the measured ANE voltage along y-direction ($V_{\text{meas}}^{\text{y}}$), that is related to $V_{\text{gen}}^{\text{y}}$ by

$$\begin{aligned} V_{\text{ANE}} &= V_{\text{meas}}^{\text{y}} \\ &= \frac{L_{\text{x}}}{w} \cdot V_{\text{gen}}^{\text{y}} \\ &\propto \frac{L_{\text{x}}}{w} \cdot \frac{\mu \cdot S_{\text{ANE}} \cdot P}{L_{\text{x}}} \end{aligned}$$

$$V_{\text{ANE}} \propto \frac{\mu \cdot S_{\text{ANE}} \cdot P}{w} \tag{S2}$$

Thus, we see that the magnitude of the ANE signal only depends on the total incident power and the wire width, and is independent of the heat flux area. To verify this experimentally, we heat the magnetic wire with constant power over areas of different dimensions. This is achieved by focusing a laser beam using objective lenses with two different numerical apertures (NA) of 0.4 and 0.7. Since the diffraction-limited spot size of the focused laser beam is inversely proportional to the NA, these yield different heat flux areas, as schematically depicted in Figure S4b. Although, the heat flux area is larger for $NA = 0.4$ relative to $NA = 0.7$, ANE line scans across the magnetic wire show that the magnitude of the ANE signal is identical for $NA = 0.7$ and $NA = 0.4$ (Figure S4b), confirming our analytical model.

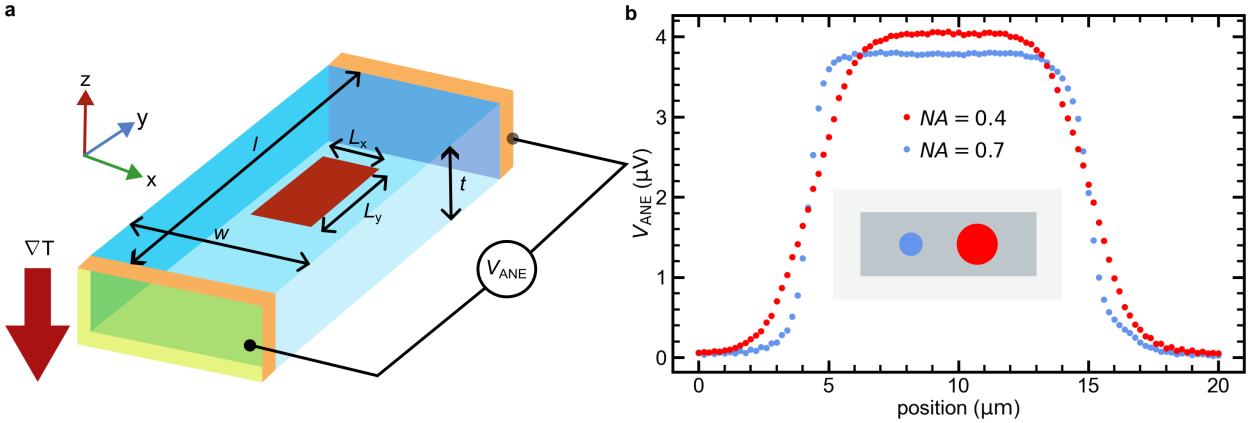

Figure S4: (a) Schematic showing a magnetic wire subjected to a heat flux in a square area (red). (b) ANE line scans across a magnetic wire using these different objective lenses. The inset schematically shows the relative size of the focal spot for $NA = 0.4$, and $NA = 0.7$. The heat flux area created by the $NA = 0.4$ objective is larger than that created by the $NA = 0.7$ objective.

S5 Laser heating induced temperature gradient

We use finite element modelling simulations in COMSOL multiphysics to estimate the spatial and temporal temperature distribution in a 15 nm CoFeB film subjected to laser induced heating (Figure S5a). The laser beam intensity is modelled as a Gaussian , with full width at the half maxima (FWHM) $= 2.355\sigma$. For an input laser power of P (in mW) on the sample, the intensity distribution is,

$$I(r) = \frac{P}{2\pi \cdot \sigma^2} \cdot e^{-\left(\frac{r^2}{2\sigma^2}\right)} \qquad [\mathrm{W/m^2}]$$

Taking the reflection coefficient of our CoFeB films to be $1 - \alpha$, a fraction of the beam (αP) passes in the z-direction through the film and is attenuated exponentially. Thus we have

$$I(r, z) = \frac{\alpha \cdot P}{2\pi \cdot \sigma^2} \cdot e^{-\left(\frac{r^2}{2\sigma^2} + \frac{z}{z_0}\right)} \qquad [\mathrm{W/m^2}]$$

where $z_0 = 10\,\text{nm}$ is the skin depth of the CoFeB film. The laser beam intensity absorbed within a thickness δz is $\frac{dI}{dz} \cdot \delta z$. Thus, the absorbed laser beam energy per unit volume is $\frac{dI}{dz}$,

$$Q(r,z) = \frac{dI}{dz} = \frac{\alpha \cdot P}{2\pi \cdot \sigma^2 z_0} \cdot e^{-\left(\frac{r^2}{2\sigma^2} + \frac{z}{z_0}\right)} \qquad [\mathrm{W/m^3}]$$

This acts as a local heat source, which is modulated at the frequency $f = 1/T$ and can be modelled as $\sin^2\left(\frac{2\pi t}{T}\right)$. The spatial and temporal dependence of the heat source can therefore be written as:

$$Q(r,z,t) = \frac{\alpha \cdot P}{2\pi \cdot \sigma^2 \cdot z_0} \cdot e^{-\left(\frac{r^2}{2\sigma^2} + \frac{z}{z_0}\right)} \cdot \sin^2\left(\frac{2\pi t}{T}\right) \qquad [\mathrm{W/m^3}]$$

The CoFeB film temperature rises due to this heat source and subsequently the absorbed heat diffuses through to the MgO substrate. We use the heat conduction module in COMSOL to solve this diffusion equation numerically. We simulated two different scenarios, representing the SANE ($\alpha \cdot P = 0.35 \times 5\,\text{mW} = 1.75\,\text{mW}$, FWHM $= 2.355\sigma = 750\,\text{nm}$, $f = 600\,\text{Hz}$) and NF-SANE ($\alpha \cdot P = 0.05 \times 7 \times 10^{-2}\,\text{mW} = 3.5 \times 10^{-3}\,\text{mW}$, $2.355\sigma = 20\,\text{nm}$, $f = 580\,\text{kHz}$) cases. In the SANE scenario, the temperature of the film directly underneath the laser spot rises to $\approx$314 K at time $= T/4$, when the laser power is at a maximum. A line graph along the z direction shows a monotonous decrease of the temperature, resulting in a net gradient of $\approx$0.13 K across the 15 nm film Figure S5b. Results of the similar nature are obtained in the NF-SANE case as well.

The measured ANE signal is proportional to the average temperature gradient across the film $\nabla T_\mathrm{z} = \frac{T_\mathrm{top} - T_\mathrm{bottom}}{t}$. To investigate the temporal distribution of the thermal gradient, ∇T_z is plotted against time. It follows the laser intensity modulation in both cases (Figure S5c). This demonstrates that we can generate a modulated temperature gradient at both, the frequency of the optical chopper (used in the SANE microscope) and the vibrating AFM tip (used in the NF-SANE microscope).

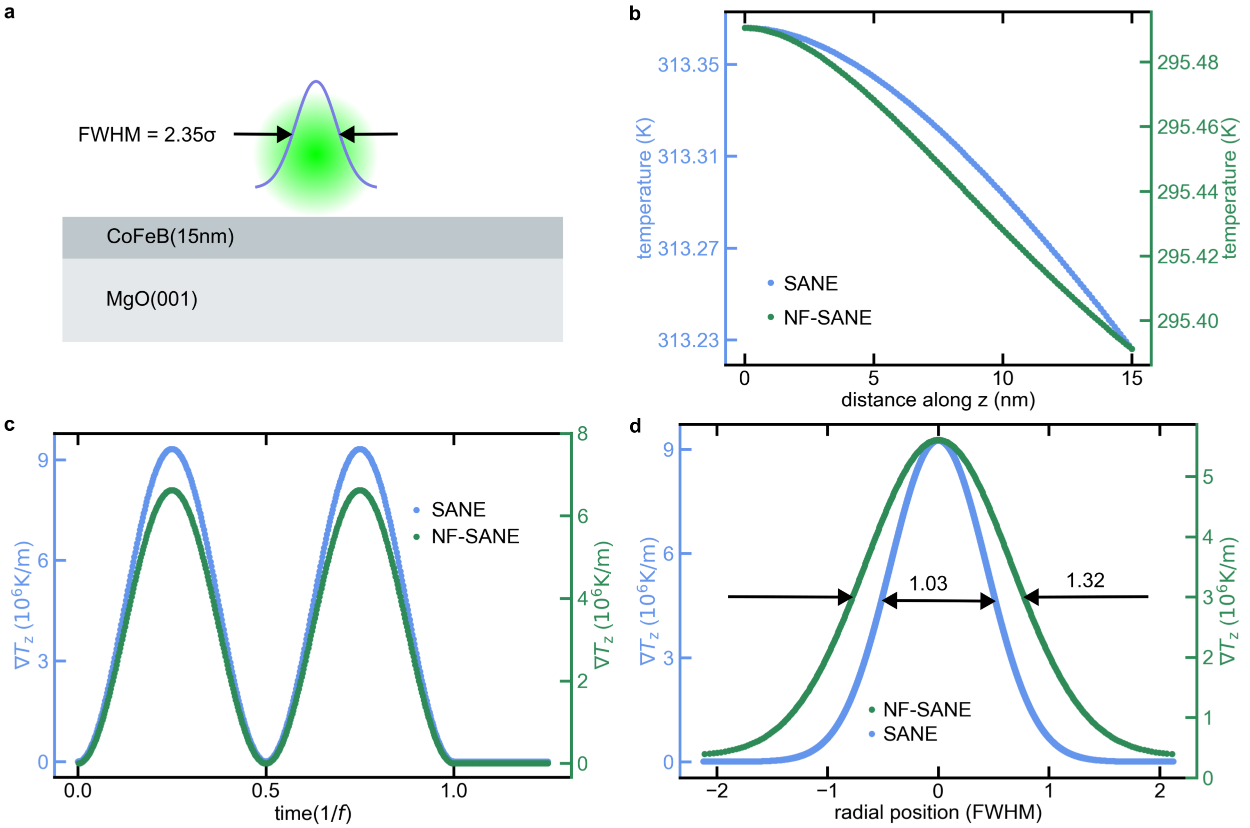

Figure S5: (a) Schematic showing a metallic film on a transparent substrate illuminated by a Gaussian laser beam. (b) Line graph of temperature across the film thickness at time $= T/4$. (c) Time dependence of the average temperature gradient across the film thickness ∇T_{z}. (d) Radial distribution of ∇T_{z}. The length scale on the horizontal axis is normalised to the FWHM of the laser intensity distribution. In (b-d), left (blue) axis shows the simulation results for the SANE scenario, right (green) axis for the NF-SANE case.

We also study the spatial distribution of ∇T_{z} across the plane of the film. The radial distribution of ∇T_{z} underneath the laser spot is fitted to a Gaussian function and the fit is plotted in (Figure S5d). We find that in the SANE case, FWHM of ∇T_{z} is only 3% higher relative to FWHM of the laser intensity distribution. Similarly, for the NF-SANE case, the gradient distribution spread is 32% higher relative to laser intensity distribution. Thus a ∇T_{z} arises only in the area directly underneath the laser spot.

The spatial distribution of the film temperature in the x direction is shown in (Figure S6). The derivative of this curve gives the in-plane (IP) temperature gradient along the x-direction (∇T_{x}). We find that this gradient is antisymmetric and that the net ∇T_{x} across the

laser beam profile is zero. However, when only part of the laser beam is considered, the net ∇T_{x} can be non-zero and comparable in magnitude to that of the out-of-plane temperature gradient.

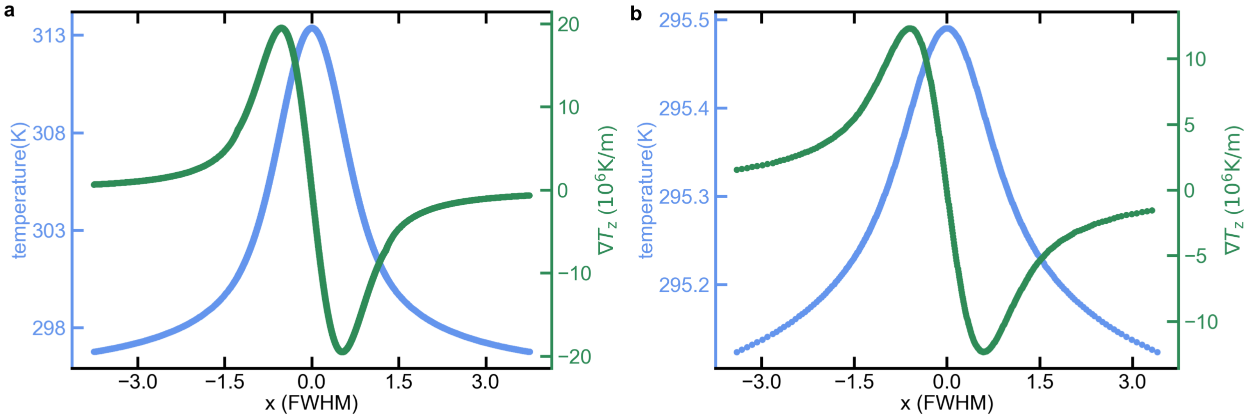

Figure S6: (a) Temperature underneath the laser spot in the SANE microscope setup at time $= T/4$: line profile of the film temperature along the x-direction (blue, left axis), and IP temperature gradient ∇T_{x} (green, right axis)(b) Same for the NF-SANE case. The length scale on the horizontal axis is normalised to the FWHM of the laser intensity distribution.

S6 Intensity distribution of the focused laser beam

We determine the intensity distribution ($I(r)$) of the focused laser beam utilised in SANE microscope. This is obtained by scanning an edges of a thin gold film under the laser focal spot (Figure S7a), and measuring the intensity of the reflected beam (I_{ref}). I_{ref} as a function of edge position (x) can be written as

$$I_{\mathrm{ref}}(x) = \int_{-\infty}^{x} r_1 I(r)dr + \int_{x}^{\infty} r_2 I(r)dr$$

Where, r_1 and r_2 are the reflection coefficient of the transparent substrate and the gold film respectively. Defining $\int I(r)dr = I^{\mathrm{int}}(r)$, we have:

$$I_{\mathrm{ref}}(x) = (r_1 - r_2) \cdot I^{\mathrm{int}}(x) + constant$$

Taking the derivative of above equation give,

$$\frac{\mathrm{d}I_{\mathrm{ref}}(x)}{\mathrm{d}x} = (r_1 - r_2) \cdot \frac{\mathrm{d}I^{\mathrm{int}}(x)}{\mathrm{d}x} = (r_1 - r_2) \cdot I(x)$$

$$I(x) \propto \frac{\mathrm{d}I_{\mathrm{ref}}(x)}{\mathrm{d}x} \tag{S3}$$

Thus we see that laser intensity distribution $I(x)$ is proportional to derivative of the reflectivity line scan. Figure S7b shows reflectivity measurement as the edge is scanned across the focused laser beam spot, the line scan is fitted to a function $f(x) = a + bx + erf(x)$. Where, $erf(x)$ is an error function. The derivative of this line scan indicates that the laser intensity distribution is a Gaussian distribution with a FWHM $= 736 \pm 22\,\mathrm{nm}$.

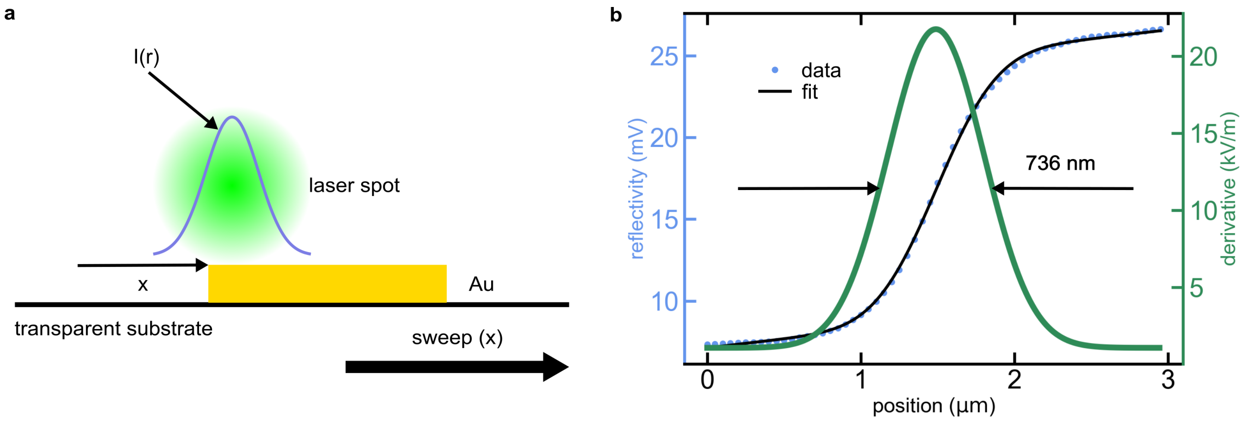

Figure S7: (a) Schematics showing an edge of a gold thin film being swept across a laser beam focal spot. (b) Plot of reflectivity against the laser spot position (blue, left axis). The line scan is fitted to an error function. Spatial derivative of the fitted curve is shown in green, right axis.

S7 Generalised expression for ANE Signal in ANE microscope

Consider a magnetic slab of the dimension $l \times w \times t$ (Figure S4a) illuminated by a laser beam. A temperature gradient ∇T_{x} and ∇T_{z} induces an electrical response along the slab

length (y-direction) due to ANE,

$$E_{\mathrm{ANE}} = \mu \cdot S_{\mathrm{ANE}} \cdot (m_{\mathrm{x}} \nabla T_{\mathrm{z}} + m_{\mathrm{z}} \nabla T_{\mathrm{x}})$$

Since the gradient is non-uniform under the laser spot, we divide the entire slab into cubes of dimension $dx \times dy \times dz$ so that within these cubes, the gradient can be assumed to be constant. The resulting voltage is given as follows:

$$V_{\mathrm{ANE}} = E_{\mathrm{ANE}}\, dy = \mu \cdot S_{\mathrm{ANE}} \cdot (m_{\mathrm{x}} \nabla T_{\mathrm{z}} + m_{\mathrm{z}} \nabla T_{\mathrm{x}})\ dy$$

These stacks of the cubes act as batteries conducting in parallel along x and z direction and in series along y direction. The net voltage averages out in x and z directions and adds up in y-direction,

$$V_{\mathrm{ANE}} = \frac{\mu \cdot S_{\mathrm{ANE}}}{w \cdot t} \cdot \left(\int_0^w \int_0^l \int_o^t m_{\mathrm{x}} \nabla T_{\mathrm{z}}\, dxdydz + \int_0^w \int_0^l \int_o^t m_{\mathrm{z}} \nabla T_{\mathrm{x}}\, dxdydz \right).$$

Assuming m_{x} and m_{z} to be uniform across the film thickness of a few nanometers, we have,

$$\int_0^t m_{\mathrm{x}} \nabla T_{\mathrm{z}}\, dz = m_{\mathrm{x}} \int_0^t \nabla T_{\mathrm{z}}\, dz = m_{\mathrm{x}} \cdot (T_{\mathrm{top}} - T_{\mathrm{bottom}})$$

The temperature across the film is nearly identical, for example 313.35 K and 313.23 K in Figure S5b. ∇T_{x} can be assumed to be nearly constant across the film thickness, giving $\int_0^t m_{\mathrm{z}} \nabla T_{\mathrm{x}}\, dz = m_{\mathrm{z}} \nabla T_{\mathrm{x}} t$. Therefore,

$$V_{\mathrm{ANE}} = \frac{\mu \cdot S_{\mathrm{ANE}}}{w} \cdot \left(\frac{1}{t} \cdot \int_0^w \int_0^l m_{\mathrm{x}} \cdot (T_{\mathrm{top}} - T_{\mathrm{bottom}})\, dxdy + \int_0^w \int_0^l m_{\mathrm{z}} \nabla T_{\mathrm{x}}\, dxdy \right) \qquad \text{(S4)}$$

S8 Spatial distribution of the temperature gradient

Similar to laser intensity distribution, we can determine temperature gradient distribution $g(r)$ by scanning a domain wall across the heat source spot (Figure S8a). Resulting ANE voltage $V_{\mathrm{ANE}}(x)$ as a function of the domain wall position (x) is measured. Following the similar analysis as for the laser intensity distribution, we obtain an equation analogous to equation (S3) as following:

$$g(x) \propto \frac{\mathrm{d}V_{\mathrm{ANE}}(x)}{\mathrm{d}x} \qquad \text{(S5)}$$

The temperature gradient distribution $g(x)$ would be proportional to the ANE derivative $\frac{\mathrm{d}V_{\mathrm{ANE}}(x)}{\mathrm{d}x}$. Figure S8b shows the repetition of the measurement shown in the main text Figure 4c. A line scan across the the vortex is shown in Figure S8c. The line scan is fitted to $f(x) = a + bx + erf(x)$. It's derivative indicates a temperature gradient distribution $g(x)$, which is a Gaussian function with a FWHM $= 76\,\mathrm{nm}$.

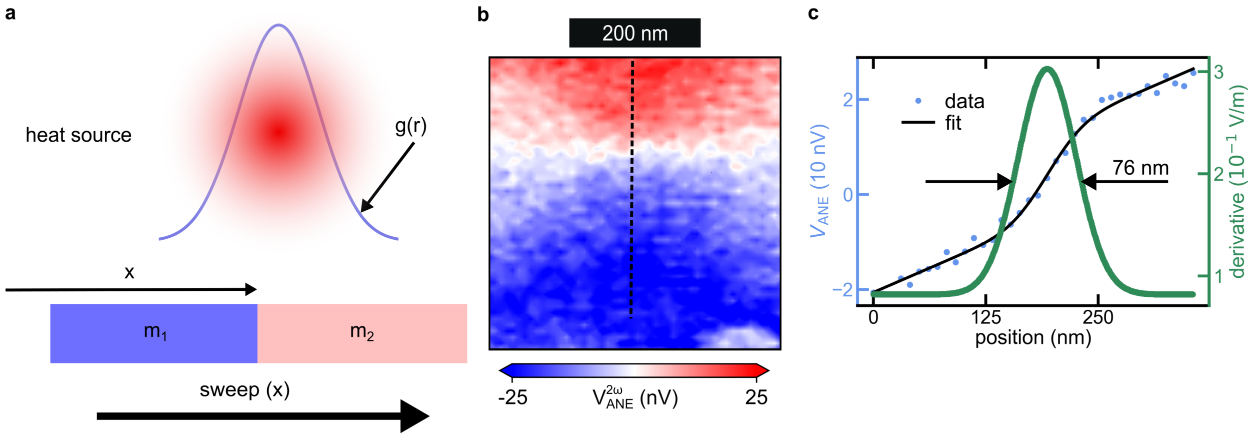

Figure S8: (a) Schematics showing a magnetic domain wall across a heat source.(b) Repetition of the measurements in the main text Figure 4c. (b) ANE line scan in the region shown by the dotted rectangle in (b). The line scan is fitted to an error function. The right, green axis shows the derivative of the fitted line.

S9 Magnitude of the OOP temperature gradient

We compute the magnitude of OOP temperature gradient ∇T_{z} based on V_{ANE} in a saturated IP magnetised CoFeB film ($m_{\text{x}} = 1, m_{\text{z}} = 0$). Assuming ANE coefficient to be that of a typical ferromagnet ($\mu S_{\text{ANE}} = 1\,\mu\text{V/K}$), equation (S4) simplifies to the following:

$$V_{\text{ANE}} = \frac{1\,\mu\text{V/K}}{w} \int_0^w \int_0^l \nabla T_{\text{z}}\, dxdy$$

We showed in the previous section that ∇T_{z} is a Gaussian function function of x, y with standard deviation of $\sigma = \frac{FWHM}{2.35}$. Defining to ∇T_{z}^0 to be the gradient at the centre of the of the laser spot,

$$V_{\text{ANE}} = \frac{1\,\mu\text{V/K}}{w} \nabla T_{\text{z}}^0 \int_0^w \int_0^l e^{\frac{-(x^2+y^2)}{2\sigma^2}}\, dxdy$$

Since l, w are much larger than σ, limits in the integration can be considered as $-\infty$ to ∞. Then the value of the integral is $2\pi\sigma^2$,

$$V_{\text{ANE}} = \frac{1\,\mu\text{V/K}}{w} \cdot \nabla T_{\text{z}}^0 \cdot 2\pi\sigma^2 \tag{S6}$$

In the case of SANE, an ANE signal $4\,\mu\text{V}$ is observed in a $10\,\mu\text{m}$ wide wire. Here, $\sigma = \frac{FWHM}{2.35} = \frac{760\,\text{nm}}{2.35}$ is $\approx 324\,\text{nm}$. Substituting these value in equation (S6) gives $\nabla T_{\text{z}}^0 \approx 6 \times 10^6\,\text{K/m}$. Similarly, for the NF-SANE case, $V_{\text{ANE}} = 40\,\text{nV}, w = 2\,\mu\text{m}, \sigma = 32\,\text{nm}$ gives, $\nabla T_{\text{z}}^0 \approx 12 \times 10^6\,\text{K/m}$.

S10 NF-SANE spatial resolution with the domain wall in PMA wire

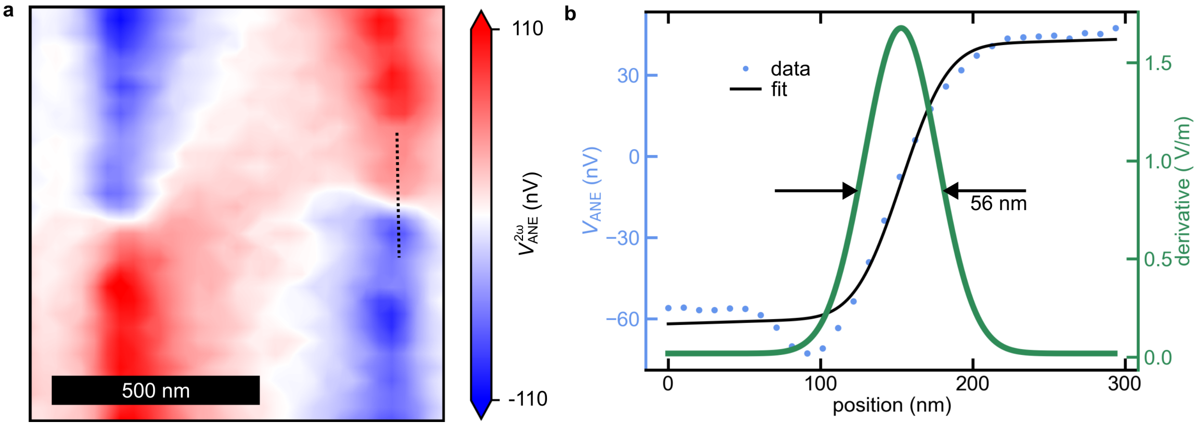

Figure S9: (a) Figure 5d in the main text. (b) ANE line scan across a magnetic domain wall shown by the dotted line in (a). The derivative of the fit to the line scan is shown on the right, green axis.

S11 Micromagnetic simulations

For the micromagnetic simulation using MuMax3, CoFeB magnetic structures were discretized in cells of size 512 nm^3, thus the step size (8 nm) is less than the exchange length (λ_{exc} =8.6 nm) of $Co_{20}Fe_{60}B_{20}$. We have taken $M_S = 65\,kAm^{-1}$, in-plane magnetic anisotropy constant $K = 2.02\,kJm^{-3}$, exchange constant A =20 pJm^{-1} , damping coefficient $\alpha = 0.03$. We initialed with random magnetization and then applied the exponentially decreasing oscillating magnetic field to create a vortex ground state. The Runge-Kutta (RK45) method was used for the relaxation process.

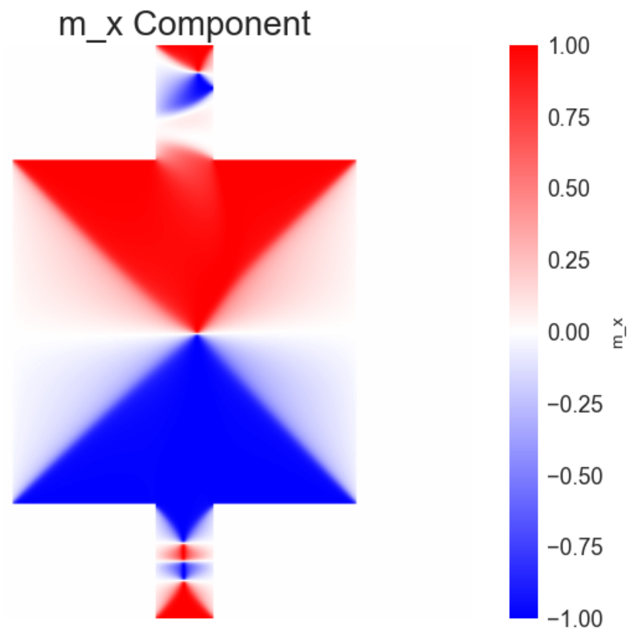

Figure S10: Micromagnetic simulation for the the device discussed in the main text figure.4.

S12 Magnetic characterization with SANE microscopy

We demonstrate that the SANE microscope can be utilised for characterizing magnetic properties, for example a hysteresis loop measurement. We do so by scanning the laser over a $15\,\mu m \times 5\,\mu m$ area and averaging V_{ANE} at different applied field strengths, as shown in Figure S11. For this measurement, a 15 nm thick CoFeB film with width $w = 14\,\mu m$ and length $l = 25\,\mu m$ is chosen. The wider wire reduces shape anisotropy and provides reasonable coercive field to observe a hysteresis.

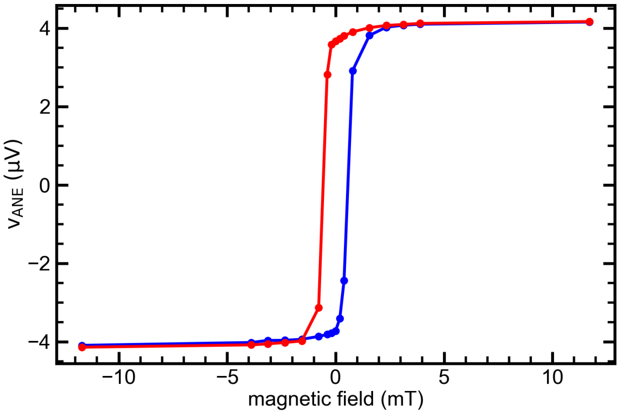

Figure S11: Hysteresis measurement of the 15 nm thick CoFeB film with SANE microscopy

S13 Magnetic property of the Co/Ni film

Here we show hysteresis measurement for the Co/Ni multi stack PMA film. The device made out of this film were utilized to show ANE domain imaging in out-of-plane magnetized sample. The measurements are performed with a vibrating-sample magnetometer.

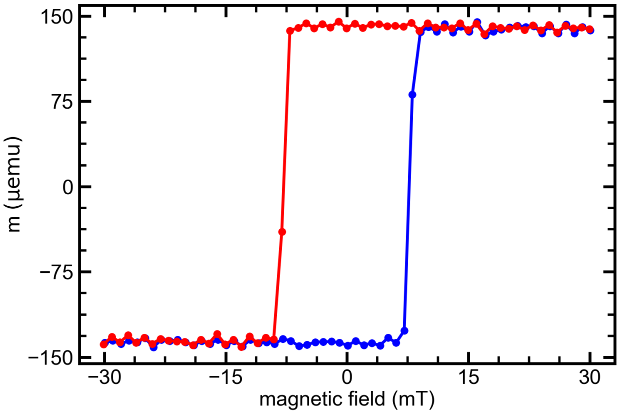

Figure S12: Hysteresis measurement of the Co/Ni film.
